# Protein lactylation in kidney diseases

**DOI:** 10.3389/fcell.2025.1533175

**Published:** 2025-08-13

**Authors:** Yelei Xu, Xinming Li, Zhiguo Mao, Cheng Xue

**Affiliations:** ^1^ Naval Medical University (Second Military Medical University), Shanghai, China; ^2^ Division of Nephrology, Shanghai Changzheng Hospital, Second Affiliated Hospital of Naval Medical University (Second Military Medical University), Shanghai, China

**Keywords:** lactate, lactylation, post-translational modification, chronic kidney disease, acute kidney injury, diabetic kidney disease

## Abstract

Post-translational modifications (PTMs) regulate protein function and structure through covalent modifications, participating in various physiological and pathological processes. Lysine lactylation is an emerging PTM discovered in recent years, which regulates gene expression, metabolism, and cell signaling by adding a lactyl group to lysine residues. As a by-product of glycolysis, lactate not only influences cellular metabolism but also contributes to the development and progression of kidney diseases through lactylation. This review focuses on the discovery and regulatory mechanisms of lysine lactylation, particularly its role in kidney diseases such as acute kidney injury, chronic kidney disease, and diabetic kidney disease. Lactylation influences the metabolic state, inflammatory response, and fibrosis of renal cells by modulating protein function and key gene transcription. With continued research, lactylation is expected to become a novel target for understanding the metabolic-epigenetic regulation of kidney diseases and could offer new strategies for treatment.

## 1 Introduction

Post-translational modifications (PTMs) are crucial regulatory mechanisms that affect protein function, stability, localization, and interactions (Ramazi and Zahiri, 2021). They play a significant role in numerous physiological and pathological processes, including signal transduction, gene expression, and metabolic regulation. Kidney diseases (KD), characterized by various forms of kidney dysfunction including acute kidney injury (AKI) and chronic kidney disease (CKD), are complex conditions influenced by genetic, environmental, and metabolic factors (Fontecha-Barriuso et al., 2018). Research has found that PTMs play a significant role in the pathogenesis of various KD, including renal cell carcinoma, AKI, DKD, renal fibrosis, and lupus nephritis. The histone methyltransferase enhancer of zeste homolog 2 (EZH2) regulates ferroptosis through histone methylation, thus promoting calcium oxalate-induced renal injury (Yan et al., 2024). Furthermore, EZH2 is also upregulated in sepsis-induced AKI (SA-AKI), increasing apoptosis and inflammatory responses in renal tubular epithelial cells (TECs) and aggravating renal tubular injury (Li B. et al., 2023). Autosomal dominant polycystic kidney disease (ADPKD) is one of the most common hereditary kidney diseases globally and a major cause of end-stage kidney disease (ESKD). Recent research has discovered that aberrant methylation in ADPKD is associated with histone and non-histone methylation, participating in the regulation of cell cycle, ciliogenesis, and cyst growth (Xu et al., 2022). Moreover, histone deacetylase (HDAC) modulates the expression of the polycystic kidney disease-1 gene (*Pkd1).* HDAC inhibitors can reduce cyst formation in *Pkd1*-deficient mice (Van Bodegom et al., 2006) and delay the impairment of renal function in *Pkd2*-deficient mice (Xia et al., 2010). The impact of PTMs on kidney diseases may be protective or harmful, and exploring its specific mechanisms could help find a new therapeutic target for kidney diseases. Recently, novel PTMs, such as lactylation, have gained attention for their potential involvement in regulating protein function in kidney diseases, offering new insights into disease mechanisms and potential therapeutic targets (Liu et al., 2023).

Protein lactylation is a newly identified PTM characterized by the covalent attachment of a lactyl group to lysine residues in proteins (Izzo and Wellen, 2019). This modification is derived from lactate, a by-product of glycolysis, and plays a key role in regulating various biological processes,. Lactate serves not only as a crucial end product of glycolysis but also as a precursor for lactylation. In recent years, protein lactylation has been linked to several diseases, including kidney disease, and is emerging as a novel target for understanding the regulatory mechanisms of metabolic epigenetics. The comparison of lactylation with other common PTMs, such as methylation, acetylation, SUMOylation, phosphorylation, crotonylation, and neddylation, is presented in Table 1.

**TABLE 1:** Identification of various types of PTMs.

PTM	Modification feature	Group donor	Tool enzyme	Biochemical functions	Involvement in kidney diseases
Lactylation	Addition of lactyl groups to lysine residues	Lactate	Histone acetyltransferase, Histone deacetylase	Promoting gene expression, participating in metabolic regulation and cell signaling	AKI, CKD, DKD
Methylation	Addition of methyl groups to lysine or arginine residues	S- Adenosylmethionine, etc.	Histone methyltransferase, Histone demethylases	Inhibiting or promoting gene expression and regulating protein stability	AKI (Li B. et al., 2023), kidney fibrosis (Zhou et al., 2023), ADPKD^a^ (Lv et al., 2024)
Acetylation	Addition of acetyl groups to lysine residues	Acetyl-CoA	Histone acetyltransferase, Histone deacetylase	Promoting gene expression, regulating chromatin tightness and protein stability	AKI (Liang et al., 2024), kidney fibrosis (Zhang et al., 2021), SA-AKI (An et al., 2023), ADPKD (Fan et al., 2012)
Palmitoylation	Addition of palmitoyl groups to cysteine residues	Palmitoyl-CoA	Palmitoyltransferas, Acyl-Protein Thioesterase	Regulating signaling pathways, cytoskeleton localization and protein membrane localization and stability	Kidney fibrosis (Gu et al., 2023)
Phosphorylation	Addition of phosphate groups to serine, threonine or tyrosine residues	ATP	Protein kinase, Phosphatase	Regulateing signaling pathways, gene expression and promoting chromatin remodeling	DKD (Feng, 2020), kidney fibrosis (Yang et al., 2023)
Ubiquitination	Addition of ubiquitin molecules to lysine residues	Ubiquitin	UBA1^b^, UBA2, E3 ubiquitin ligase, deubiquitinating enzyme	Promoting the degradation of proteins and regulating protein homeostasis	AKI (Ma Y. et al., 2024), DKD (Li S. et al., 2023), kidney fibrosis (Wei et al., 2024), HRD^c^ (Zhang et al., 2022), PKD (Zhong et al., 2024)
SUMOylation	Addition of SUMO proteins to lysine residues	SUMO proteins	SAE1^d^, SUMO E2 enzyme, SUMO E3 ligase	Regulating protein stability and cellular stress response	DKD (Lizotte et al., 2023), AKI (Zhu et al., 2023)
Crotonylation	Addition of crotonyl groups to lysine residues	Crotonyl-CoA	HAT, HDAC	Activating gene expression and regulating energy metabolism	Renal fibrosis (Li Y. et al., 2024), ADPKD (Dang et al., 2022)

^a^ Autosomal Dominant Polycystic Kidney Disease.

^b^ Ubiquitin-activating enzyme.

^c^ Hypertensive Renal Disease.

^d^ SUMO-activating enzyme subunit1.

## 2 Metabolism of lactate

Lactate was once thought to be a metabolic waste product of glycolysis during hypoxia with deleterious effects. However, more and more studies have proved that lactate not only acts as an energy supply for cells, but also participates in numerous pathophysiological processes such as gluconeogenesis, cell signaling, and immunomodulation (Li et al., 2024b).

In the process of glucose metabolism, glucose enters the cell through transport proteins and produces pyruvate under the catalysis of key enzymes, such as hexokinase (HK), phosphofructokinase-1 (PFK-1), and pyruvate kinase (PK). Under normoxia, pyruvate enters into the mitochondria and is catalyzed by pyruvate dehydrogenase (PDH) into acetyl coenzyme A (Acetyl-CoA) which participates in oxidative phosphorylation (OXPHOS) to generate ATP for cellular process; while under hypoxic conditions, the mitochondrial OXPHOS pathway is inhibited, and pyruvate is catalyzed by lactate dehydrogenase (LDH) to generate lactate. The clearance of lactate in cells occurs mainly through two pathways. In one pathway, lactate participates in hepatic and kidney gluconeogenesis as a substrate. In the other pathway, lactate is oxidized to pyruvate, which shuttles into mitochondria through monocarboxylate transporters (MCTs) to participate in the tricarboxylic acid cycle and generate ATP for cellular metabolism (Li et al., 2024b). At the beginning of the 20th century, Warburg (Warburg, 1956) found that in the presence of sufficient oxygen, tumor cells would still preferentially generate lactate via glycolysis after uptaking glucose and meanwhile inhibit the metabolism of pyruvate in mitochondria. This shift in metabolism contributes to optimizing energy utilization of tumor tissues, promoting their proliferation and invasion, which is the famous ‘Warburg’ effect. This phenomenon occurs not only in tumors but also in high proliferation activity tissues such as angiogenesis, inflammatory response, damage repair, and immune response (Vander Heiden et al., 2009). It has been found in recent years that lactate also acts as a precursor of lactylation and participates in regulating key cellular activities.

## 3 Protein lactylation

In 2019, Zhang et al. found that there exists lactylation of histone lysine in tumor cells and macrophages. Lactylation is a novel form of PTM deriving from lactyl-coenzyme A (Lactyl-CoA). It was further validated that histone lactylation influences chromatin and gene expression through epigenetic mechanisms. When an inflammatory response is triggered (Zhang et al., 2019), pro-inflammatory M1 macrophages are rapidly activated and release pro-inflammatory cytokines which induce the expression of inflammatory genes such as Type 2 nitric oxide synthase. Excess lactate is produced and accumulates as a result of metabolic reprogramming in M1 macrophages. In the late stage of the inflammatory response, excess lactate promotes histone lactylation. H3K18la (lactylation of histone 3 lysine 18, K for lysine) is enriched at the promoter of M2-like genes such as arginase 1, and promotes its transcription which contributes to the shift of macrophage phenotype from M1 to anti-inflammatory M2 and helps to repair the damage due to the inflammatory response. Since then, this research has opened a new chapter in the exploration of lactylation, offering a fresh perspective on the relationship between metabolic reprogramming and epigenetic modifications.

### 3.1 Histone lactylation

Histones are basic DNA-binding proteins found in chromosomes. The nucleosome, the fundamental repeating unit of chromatin, consists of 146 bp DNA wrapped around an octamer of the core histones (H2A, H2B, H3, and H4), along with the linker histone H1 (Nitsch et al., 2021). Histone lactylation primarily occurs on lysine residues. It modifies chromatin structure and function by altering the net charge of histones and influencing the binding affinity between histones and DNA. This leads to chromatin relaxation and easier recognition and binding of transcription factors, thereby activating gene expression (Luger et al., 2012).

Lactylation on the same site may exert diverse pathophysiological effects through downstream signaling on anti-inflammation, angiogenesis, and tumor proliferation and invasion. H3K18la can not only increase the level of YTH N6-Methyladenosine RNA Binding Protein F2, the reader of methylation to drive ocular melanomagenesis (Yu et al., 2021), but also activate the transcription of platelet-derived growth factor receptorβ in von Hippel-Lindau-mutated clear cell renal cell carcinomas which in turn increases lactylation levels and forms an oncogenic positive feedback loop (Yang J. et al., 2022). Moreover, H3K18la is also linked to the severity of septic shock (Chu et al., 2021) and SA-AKI (Qiao et al., 2024), the promotion of embryo implantation and its growth (Li J. et al., 2024), and the macrophage phenotype switch from M1 to M2 (Zhang et al., 2019). Apart from H3K18la, more and more histone lactylation sites are found to be involved in diverse diseases. For example, glycolysis aggravates microglia dysfunction in Alzheimer’s disease through the H4K12la/PKM2 positive feedback loop (Pan R. Y. et al., 2022). Triterpenoid antitumor compound demethylzeylasteral suppresses the proliferation of hepatocellular carcinoma (HCC) stem cells and impedes HCC progression by reducing H3K9la and H3K56la (Pan L. et al., 2022).

### 3.2 Non-histone lactylation

Non-histone lactylation mainly occurs on lysine residues as well. It regulates protein function by affecting steric hindrance, conformational changes, and charge neutralization (Yang et al., 2024). For example, it alters molecular interactions, enzyme activity, and subcellular localization of the proteins.

So far, the majority of the research conducted on lactylation has focused on histone lactylation instead of non-histone lactylation. Nonetheless, several studies have demonstrated the importance of non-histone lactylation in regulating gene transcription and signal transduction. Luo et al. found that (Luo et al., 2022) lactate imported to cells by MCT1 helps to stabilize hypoxia inducible factor-1α (HIF-1α) via its lactylation which enhances the transcription of cell migration-inducing protein to promote angiogenesis and vasculogenic mimicry in prostate cancer. After cerebral infarction, lactate accumulates in ischemic and hypoxic tissues and induces lymphocyte cytosolic protein 1 (LCP1) lactylation, which can stabilize LCP1, thus aggravating neuron injuries and the progression of cerebral infarction (Zhang W. et al., 2023).

### 3.3 Two pathways of lysine lactylation

**FIGURE 1:**
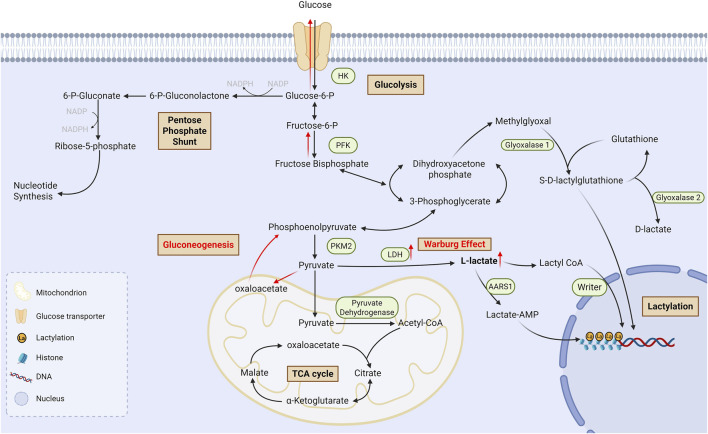
Process of lactate metabolism and lysine lactylationGlucose undergoes glycolysis to produce L-lactate and D-lactate. There are also two pathways of lactylation: L-lactylation and D-lactylation. L-lactate is conversed into lactyl-CoA in a CoA-transfer reaction, and then the lactyl group is transferred to lysine residues which is catalyzed by non-specific writers; Moreover, AARS1, a specific lactyltransferase, could bind to lactate and catalyzes the formation of lactate-AMP, followed by transfer of lactate to the lysine residue. On the other hand, D-lactylation utilizes S-D-lactoylglutathione as the donor of lactyl group and indirectly delivers lactyl group through a non-enzymatic process. TCA cycle: Tricarboxylic acid cycle; AMP: Adenosine monophosphate; AARS1: Alanyl-tRNA synthetases 1.

There are 2 enantiomers of lactate in the human body, L-lactate and D-lactate, the former of which is predominant. Meanwhile, there are two pathways of lactylation: L-lactylation and D-lactylation (Xu B. et al., 2024), also known as direct lactylation and indirect lactylation (Figure 1). L-lactylation is an enzymatic reaction which is mostly catalysed by non-specific acetyltransferases utilizing lactoyl CoA as the donor of lactyl group; recently it has been found that alanyl-tRNA synthetases 1 (AARS1), a specific lactyltransferase, binds to lactate and catalyzes the formation of lactate-AMP, followed by transfer of lactate to the lysine residue (Zong et al., 2024). On the other hand, D-lactylation, also referred to as non-enzymatic lactylation, utilizes S-D-lactoylglutathione (LGSH) as the donor of lactyl group and indirectly delivers lactyl group through a non-enzymatic process (Gaffney et al., 2020). L-lactylation is the predominant pathway in cells (Zhang D. et al., 2024), which is also the main pathway influenced by glycolysis since it dynamically regulates lactyl CoA.

### 3.4 Regulatory mechanisms of lactylation

The process of lactylation is influenced by lactate metabolism and transport, acetyltransferases, deacetylases, and regulatory proteins. Lactate and lactylation levels are decreased when glycolysis inhibitors are used but increased when inhibitors of mitochondrial electron-transport-chain are used (Qiao et al., 2024). In addition, gene expression can in turn affect lactylation and form a positive/negative feedback loop of epigenome-metabolome-epigenome. Other factors that may influence lactylation include neuronal excitability (Hagihara et al., 2021), crosstalk with other PTMs and so on. Non-enzymatic lactylation is regulated by LGSH and glyoxalase 2 (Gaffney et al., 2020). The regulatory functions of enzymes in enzymatic lactylation are primarily summarized below.

There are 3 types of lactylation-related enzymes: writers are transferases to add the lactyl group, erasers are deacetylases to remove the lactyl group, both of which dynamically regulate lactylation; the reader is a binding protein to recognize lactylation and deliver transcriptional signals (Gao et al., 2024).

Histone acetyltransferase (HAT) family functions as the writer, among which research has demonstrated that p300 and CREB-binding protein (CBP) (Yang K. et al., 2022) participate in the catalytic process of lysine lactylation, thus activating high mobility group protein box1 (HMGB1) gene expression. In contrast, inhibition of p300/CBP significantly lowers the expression of HMGB1 in macrophage cytoplasm. HDAC acts as an eraser to remove lactyl group, which can be categorized into two types. The first type includes class I, II, and IV HDAC consisting of HDAC1-11 (Xu et al., 2021), among which HDAC1-3 have the strongest activity of both de-L-lactylation and de-D-lactylation (Moreno-Yruela et al., 2022); The other type includes class III HDAC consisting of sirtuin 1–7 (SIRT), among which SIRT1-3 have strong activity of de-L-lactylation. It has been discovered (Jin et al., 2023) that SIRT3 prevents HCC from spreading by promoting the delactylation of non-histone cell cycle protein E2. Different from the non-specific enzymes mentioned above, AARS1 and AARS2 are specific lactyltransferases and sensors for lactate. They have been recently reported to promote the lactylation of p53 (Zong et al., 2024), Yes-associated proteins (Ju et al., 2024), and cyclic GMP-AMP synthetase (Li et al., 2024a), thus participating in the proliferation and immune escape of tumor cells. Meanwhile, β-alanine can bind to AARS1 competitively due to its structural similarity with lactate, reverse the process of lactylation and enhance the effect of cancer chemotherapy. Fewer studies have been done on the reader of lactylation. Hu et al. found for the first time that Brahma-related gene 1(BRG1) functions as a reader for H3K18la and participates in mesenchymal-epithelial transition (Hu et al., 2024). The summary of enzymes involved in lactylation and part of their substrate were listed in Table 2.

**TABLE 2:** Summary of enzymes involved in lactylation

Function	Enzyme	Substrate(s)	References
Writer	p300	H3K18 H4K12 High mobility group box-1 α-Myosin heavy chain	Li F. et al. (2024) Zhang and Zhang (2024) Yang K. et al. (2022) Zhang N. et al. (2023)
	GCN5	H3K18	Wang et al. (2022)
	HBO1	H3K9	Niu et al. (2024)
	CBP	H3K18 High mobility group box-1 Twist1	Li F. et al. (2024) Yang J. et al. (2022) Xu Y. et al. (2024)
	AARS	p53 Yes-associated proteins Cyclic GMP-AMP synthetase	Zong et al. (2024) Ju et al. (2024) Li et al. (2024a)
Eraser	HDAC1-3	H3K18, H4K12	Zhang and Zhang (2024)
	HDAC6	α-tubulin	Sun et al. (2024)
	SIRT1-3	H3K18 H4K16 α-Myosin heavy chain Pyruvate kinase M2 Polypyrimidine Tract Binding Protein 1 canopy FGF signaling regulator	Tsukihara et al. (2024) Fan et al. (2023) Zhang N. et al. (2023) Du et al. (2024) Zhou Z. et al. (2024) Zhang X. W. et al. (2024)
Reader	BRG1	H3K18	Hu et al. (2024)

Lactylation-related enzymes are important regulators of lactylation. Therapeutic strategies for associated diseases can be further explored by altering enzyme activity to control lactylation levels and directly regulate downstream signaling. However, the study of enzymes in lactylation is still in its early stage and more regulatory proteins need to be identified, especially the reader. In addition, given that several different PTMs share the same enzymes, whether there is any competition among them and what the specific mechanism is still need to be further explored.

## 4 Protein lactylation in kidney diseases

**FIGURE 2:**
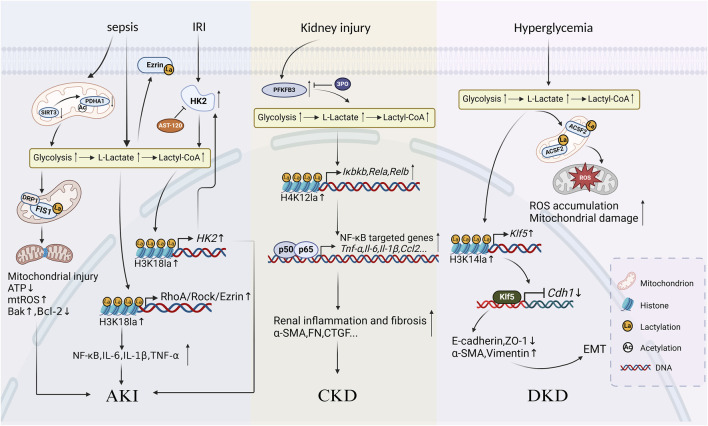
Schematic diagram of protein lactylation involved in the progression of kidney diseases Lactate accumulates and increases levels of histone or non-histone lactylation after undergoing metabolic reprogramming caused by various primary kidney injuries such as sepsis, ischemia-reperfusion injury (IRI), and hyperglycemia. Three mechanisms mainly work in the progression of kidney inflammatory responses and fibrosis: in AKI, lactylation exacerbates inflammatory responses, oxidative stress and apoptosis in renal tubular epithelial cells; in CKD, lactylation accelerates the development of renal fibrosis by activating inflammatory gene expression through the NF-κB signalling pathway; in diabetic kidney disease, lactylation promotes renal fibrosis by enhancing epithelial-mesenchymal transition.

Protein lactylation participates in the development of kidney diseases by affecting gene expression and activating or inhibiting signaling pathways (Table 3; Figure 2).

**TABLE 3:** Protein lactylation sites in various types of cells from patients with different kidney diseases.

Diseases	Cell types	Modified proteins	Modification sites	Effects	References
AKI	PTC^a^	H3	K18	Activating transcription of glycolysis key enzyme genes, exacerbating IRI cell damage	Zhou Z. et al. (2024)
SA-AKI	PTC	H3 Ezrin	K18 K263	Promoting the expression of inflammatory factor, oxidative stress, and cell apoptosis	Qiao et al. (2024)
	PTC	FIS1^b^	K20	Inducing excessive mitochondrial fission, ATP depletion, overproduction of ROS and cell apoptosis	An et al. (2023)
CKD	PTC	H4	K5, K12	Activating transcription of inflammatory genes and exacerbating kidney fibrosis	Wang Y. et al. (2024)
MAC^c^ in CKD	VSMC^d^	H3	K18	Increasing transcription of soluble phosphatase gene to release inorganic phosphorus and aggravating arterial calcification	Ma Y. et al. (2024)
DKD	PTC	ACSF2^e^	K182	Exacerbating overaccumulation of ROS and mitochondrial dysfunction	Chen et al. (2024)
	PTC	H3	K14	Inhibiting expression of cadherin and aggravating EMT	Zhang D. et al. (2024)

^a^ Proximal tubular epithelial cell.

^b^ Mitochondrial fission protein 1

^c^ Medial artery calcification.

^d^ Vascular smooth muscle cell.

^e^ Acyl-CoA, synthetase family member 2

### 4.1 Lactylation in AKI

AKI is a clinical syndrome characterized by a sudden impairment of kidney function which poses a serious threat to the patient’s life. It typically occurs during infection, trauma, and surgery, tending to be caused by various injuries such as kidney hypoperfusion, sepsis, major surgery, immune diseases, and the use of radiocontrast or nephrotoxic drugs. Poor regeneration (Hoste et al., 2018) following AKI also results in continuous deterioration in kidney function and exacerbation of kidney fibrosis, which is a risk factor for CKD and ESKD.

IRI is the primary cause of AKI (Xin et al., 2022), leading to abnormal apoptosis and inflammatory responses in renal tubular cells which is the key pathological mechanism of AKI. The low perfusion during IRI results in hypoxia, high glycolysis activity, and the accumulation of lactate and pyruvate in proximal tubular epithelial cells (PTCs). High level of lactate is an independent risk factor for the severity of SA-AKI (An et al., 2023). AST-120 (Kremezin), an oral spherical carbon adsorbent approved for CKD treatment, has been demonstrated that oral administration of it helps to alleviate IRI (Shen et al., 2021) and mitigate AKI progression. To explore whether it is related to histone lactylation, Zhou et al. established an IRI mouse model and found that the level of HK2, the key enzyme of glycolysis, increased in IRI mice while the knockout of the *Hk2* results in decreased biomarkers of PTC injury and cell death compared to the wild type (Zhou J. et al., 2024). In the presence of *Hk2*, AST-120 exerts the protective effect described above on the kidney through inhibiting glycolysis in PTC. Further mechanistic study revealed that it might be on the ground that HK2 promoted glycolysis and the production of lactate, thus inducing a significant increase in the pan-lactylation levels as well as the histone lactylation levels, especially H3K18la. Moreover, H3K18la is enriched at the promoter of *Hk2* and activates its overexpression which then promotes glycolysis in turn and forms a positive feedback loop of H3K18la-HK2-glycolysis. The loop consequently exacerbates IRI and accelerates AKI progression.

SA-AKI is usually characterized by sepsis or septic shock which is an organ dysfunction syndrome resulting from a dysregulated host response to infection and leads to progressive deterioration of kidney function (Poston and Koyner, 2019). It is strongly associated with poor clinical outcomes compared to non-septic AKI. Serum lactate is considered a prognostic biomarker of sepsis. TECs are one of the most metabolically active cells in the kidney, sensitive to sepsis-associated injury. *EZR* is a gene that encodes a protein named Ezrin, which belongs to the ezrin-radixin-moesin family. It is a crucial actin-binding protein that links membranes to actin filaments during lamellipodia formation, cell polarization, migration and signal transduction (Yin and Schnoor, 2022). Pan et al. found that H3K18la and ezrin K263la levels are increased as lactate accumulates in the cecal ligation and puncture mice and in LPS-treated PTCs (Pan R. Y. et al., 2022). Mechanistic studies have shown that H3K18la is enriched at the promoter of the Ras homolog gene family member A (*RhoA*) and increases its transcriptional activity, which aggravates inflammatory response, oxidative stress, and cell apoptosis in PTCs through the RhoA/Rho-associated kinase/Ezrin signaling axis. Moreover, increased levels of Ezrin K263la activate the Nuclear Factor kappa B (NF-κB) signaling pathway while Ezrin K263R (lysine at position 263 mutated to arginine, K for lysine) attenuates PTC injury. This study establishes a link between lactylation and SA-AKI, which may be a potential therapeutic target for regulating epigenetic modifications and metabolism in PTCs. An et al. found synergetic effects from acetylation and lactylation of non-histone in SA-AKI, providing a new idea about the role of crosstalks between multiple PTMs in kidney diseases (An et al., 2023). They found that decreased SIRT3 level in sepsis mice is accompanied by the hyperacetylation and inactivation of PDH E1 component subunit alpha (PDHA1), which leads to excessive lactate production in TECs and promotes mitochondrial fission 1 (FIS1) K20la. Mitochondria in living cells exist as a dynamic network that continuously cycle through fusion and fission events (Fenton et al., 2021). Mitochondrial fission is mainly controlled by FIS1 and the dynamin related protein 1 (DRP1), both of which exacerbates mitochondrial hyperfission, ATP depletion, reactive oxygen species (ROS) overproduction, and cell apoptosis (Egner et al., 2022). In contrast, treatment with PDHA1 activators or LDH inhibitors which increased SIRT3 levels could reverse the exacerbation of SA-AKI. Therefore, decreasing lactate production and FIS1 lactylation may be a new potential treatment to amoeliorate TEC injury and alleviate SA-AKI.

In addition, it has been found (Kim et al., 2020) that allicinol mitigates the oxidative stress, inflammatory response, and cell apoptosis in AKI induced by cisplatin at least partly through inhibiting p300-mediated epigenetic mechanisms which decreases lactylation levels of histone 3, non-histone p53 and NF-κB p65; and Tang et al. found that class I HDAC played an important role in promoting TEC protection and regeneration (Tang et al., 2014). As shared enzymes of both acetylation and lactylation, the role p300 and HDAC play in the course above may link to lactylation as well, which deserves to be further explored from the perspective of PTMs crosstalks.

### 4.2 Lactylation in CKD

The global prevalence of CKD has exceeded 10% which is a serious public health problem and needs to be solved urgently (Kovesdy, 2022). CKD is a long-term and slow-progressing disease, usually caused by other chronic diseases such as hypertension, diabetes mellitus, systemic lupus erythematosus, chronic nephritis, kidney stones, and gradually progressing AKI or usage of nephrotoxic drugs. With the fast-growing aging population and a significant increase in the prevalence of diabetes mellitus and hypertension, the incidence of CKD has been rising annually. Therefore, it is crucial to push forward early detection, management, and treatment of CKD to slow down the progression of the disease and protect kidney function at an early age.

Renal fibrosis is a common pathological feature of CKD and is characterized by renal tubular atrophy, chronic inflammation of the interstitium, myofibroblast activation, and ECM deposition (Huang et al., 2023). A significant accumulation of lactate and metabolic reprogramming of glycolysis has been observed in PTCs of CKD patients and animal models (Eymael et al., 2023), and pharmacologically suppressing glycolysis helps to prevent kidney fibrosis (Ding et al., 2017). This suggests a close correlation between lactate accumulation and progressive fibrosis in the kidney.

To further reveal the role lactylation plays in kidney fibrosis, Wang et al. explored the specific effect of 6-phosphofructo-2-kinase/fructose-2,6-biphosphatase3 (PFKFB3) on kidney fibrosis, which is a key enzyme of glycolysis (Wang Y. et al., 2024). Researchers found that PFKFB3 level significantly increases in the kidney tissues of CKD patients. Mainly localized in PTCs, it negatively correlates with kidney function while positively correlates with the degree of kidney fibrosis and levels of lactylation. Combining *in vitro* experiments for mechanistic studies, they found that PFKFB3 induces lactate accumulation and histone lactylation, especially H4K12la, through mediating metabolic reprogramming of glycolysis in PTCs. Furthermore, H4K12la is enriched at the promoter of the NF-κB pathway-related genes such as IκB kinaseβ (*Iκbkb*), *Rela*, and *Relb*, and activates target gene transcription and NF-κB signaling pathway which results in aggravating kidney inflammatory response and fibrosis. In addition, knockout of P*fkfb3* or treatment with 3PO, which is a small-molecule inhibitor of PFKFB3 can decrease levels of H4K12la and significantly suppresses the activation of NF-κB signaling pathway, remarkably attenuating kidney interstitial fibrosis and collagen deposition in IRI mice. Furthermore, recent research has demonstrated that PFKFB3 inhibitors, such as 3PO, showed a great prospect of targeting cancer cells (Kotowski et al., 2020) which is also expected to be a potential treatment for kidney fibrosis.

CKD patients are prone to develop vascular calcification due to internal imbalance of calcium and phosphorus metabolism, increased oxidative stress, and loss of calcification inhibitors (Cannata-Andía et al., 2021). Medial artery calcification (MAC) is a systematic chronic vascular disease that is common in the elderly, CKD, and diabetic patients. Different from the intimal damage in atherosclerosis, osteogenic transdifferentiation takes place in a pro-calcific CKD milieu in vascular smooth muscle cells (VSMCs) and matrix vesicles are secreted to provide a microenvironment for calcified crystals deposition, which is a key event for MAC onset and progression (Wang Z. et al., 2024). MAC is a highly risky factor for high cardiovascular morbidity and mortality in CKD patients. Ma et al. found that the level of nuclear receptor subfamily 4 group A member 3 (NR4A3) increases in CKD mice calcified aortic tissues (Ma W. et al., 2024), and the deficiency of it reduces the glycolysis and histone lactylation during calcification. Further mechanistic studies suggested that NR4A3 promotes lactate production by directly binding to the promoter of Aldolase A and Phosphofructokinase, liver type, and activating their transcription, which then increases H3K18la levels. Enriched at the promoter of Phosphoethanolamine/Phosphocholine Phosphatase 1, H3K18la activates its transcription which releases inorganic phosphorus doped into the mineral phase by hydrolyzing its substrates, thus promoting arterial calcification in CKD. Given that histone lactylation is reversible and has a close relation with kidney fibrosis and inflammation, decreasing its levels is expected to be a new target for the prevention and treatment of CKD and its complications.

### 4.3 Lactylation in diabetic kidney disease

Diabetic kidney disease (DKD) is a chronic kidney disease caused by diabetes mellitus, which is the most common microvascular complication of diabetes and can develop into ESKD (Zhang X. et al., 2024) as well. Chronic hyperglycemia brings several complex pathophysiological changes within kidney tissues, such as foot process diffuse effacement, glomerular basement membrane thickening, mesangial matrix proliferation, and glomerulosclerosis. These changes ultimately result in proteinuria, hypertension, tubular injury, decreased glomerular filtration rate, and progressive renal failure (Reidy et al., 2014). The progression of DKD is largely influenced by kidney metabolic dysregulation, mitochondrial dysfunction, and increased glycolysis, which result in greater lactate production (Lee et al., 2022). Chen et al. further explored the role metabolic reprogramming played in DKD (Chen et al., 2024). They found high levels of lysine lactylation in DKD patients and mice kidneys, most of which occur in mitochondria. DKD progression is aided by the lactylation of acyl-CoA synthetase family member 2 K182 in PTC mitochondria which further aggravates ROS hyperaccumulation and mitochondrial dysfunction.

A major factor contributing to DKD is commonly acknowledged to be the epithelial-mesenchymal transition (EMT), which is characterized by the acquisition of a mesenchymal phenotype and the loss of the epithelial cell polarity and intercellular adhesion. Zhang et al. found that glycolytic gene expression is upregulated in DKD patients and mice kidneys, accompanied by mitochondrial dysfunction and elevated lactate levels, which consequently decreases the expression of epithelial cell markers and increases the expression of mesenchymal cell markers (Zhang X. et al., 2024). Further study in mechanisms revealed that as a precursor, lactate significantly increases the pan-lactylation levels as well as H3K14la levels in TECs, which is enriched at the promoter of Krüppel-like factor 5 (*Klf5*) and facilitates its transcription. Excess KLF5 binds to the promoter of Cadherins 1 and inhibits its expression, thus promoting the progression of EMT (Li et al., 2021). Nephro-specific knockdown or pharmacological inhibition of KLF5 can alleviate EMT which is a potential strategy for DKD therapy.

## 5 Summary and prospects

Protein lactylation is increasingly recognized for its role in regulating transcriptional activity and affecting protein structure and function, with implications in diseases such as tumors, cardiovascular diseases, neurodegenerative diseases, and kidney diseases. Current treatments for kidney diseases, including AKI, DKD, and CKD, remain insufficient—using approaches like diuretics, glucocorticoids, and renal replacement therapy without fully preventing chronic kidney damage or reversing kidney insufficiency. Limited understanding of the molecular mechanisms underlying kidney disease initiation and progression has hindered the development of targeted therapies and early diagnostic approaches. Notably, lactylation links metabolism to gene regulation, providing new mechanisms and targets for treating kidney disease at the genetic level.

This review systematically elucidates the mechanisms of protein lactylation in diverse kidney diseases, including AKI, CKD, and DKD. Lactylation represents a novel metabolite-epigenetic crosstalk, regulating gene transcription through histones or non-histone modifications, which alter chromatin structure or protein function. In AKI, lactylation of FIS1 and H3K18 exacerbates mitochondrial dysfunction and inflammatory responses. In CKD, the PFKFB3-H4K12la-NF-κB axis drives renal fibrosis progression. The EMT in DKD is regulated by the H3K14la-KLF5 pathway. Lactylation could be inhibited by three strategies below: suppression of lactate production (e.g., 3PO), targeting lactylation-related enzymes (e.g., AARS1 inhibitors, HDAC/SIRT activators) and inhibition of downstream pathways (e.g., *Klf5* knockdown). However, current evidence remains confined to *in vitro* and animal experiments.

Key questions remain about protein lactylation in kidney disease. For one thing, understanding of lactylation mechanism is incomplete, including whether lactylation occurs beyond lysine residues, if it is a direct outcome of lactate accumulation in the kidney, the specific roles of lactylation-related enzymes, and how lactylation interacts with other PTMs to influence disease prognosis. For another, several barriers exist in clinical translation, including possible toxic effects on other organs because of targeting lactylation in TEC and low specificity of lactylation-related enzymes.

In conclusion, protein lactylation shows potential as a new mechanism and therapeutic target for kidney disease. Further mechanistic studies and preclinical research are essential to advance treatments for lactylation-associated kidney diseases, including exploring site-specific lactylation in kidney disease, developing kidney-targeted lactylation inhibitors, and advancing translational studies in animal models toward clinical applications.
